# Receiver aperture and multipath effects on power loss and modal crosstalk in a THz wireless link using orbital-angular-momentum multiplexing

**DOI:** 10.1038/s41598-022-18444-w

**Published:** 2022-08-18

**Authors:** Xinzhou Su, Runzhou Zhang, Zhe Zhao, Hao Song, Amir Minoofar, Nanzhe Hu, Huibin Zhou, Kaiheng Zou, Kai Pang, Haoqian Song, Brittany Lynn, Shlomo Zach, Moshe Tur, Andreas F. Molisch, Hirofumi Sasaki, Doohwan Lee, Alan E. Willner

**Affiliations:** 1 Ming Hsieh Department of Electrical and Computer Engineering, University of Southern California, Los Angeles, CA 90089 USA; 2 Naval Information Warfare Center Pacific, San Diego, CA 92152 USA; 3 School of Electrical Engineering, Tel Aviv University, 69978 Ramat Aviv, Israel; 4 NTT Network Innovation Laboratories, NTT Corporation, Yokosuka, 239-0847 Japan; 5 Ming Hsieh Department of Electrical and Computer Engineering, and Joint Appointment with Dornsife Department of Physics and Astronomy, University of Southern California, Los Angeles, CA 90089 USA

**Keywords:** Engineering, Optics and photonics

## Abstract

The channel capacity of terahertz (THz) wireless communications can be increased by multiplexing multiple orthogonal data-carrying orbital-angular-momentum (OAM) beams. In THz links using OAM multiplexing (e.g., Laguerre-Gaussian ${\mathrm{LG}}_{ \ell,p}$ beams with *p* = 0), the system performance might degrade due to limited receiver aperture size and multipath effects. A limited-size aperture can truncate the received beam profile along the radial direction. In addition, due to beam divergence, part of the beam might interact with reflectors in the environment, causing the signal to reflect and interfere at the receiver with the directly propagating part of the beam; this is known as the multipath effect. In this paper, we simulate and analyze the impact of both effects on the equality of the THz OAM link by considering a full two-dimensional (2-D) LG modal set. The simulation results show (i) a limited-size receiver aperture can induce power loss and modal power coupling mainly to LG modes with the same *ℓ* but *p* > 0 for directly propagated OAM beams; (ii) the multipath effect can induce modal power coupling across multiple 2-D LG modes, which leads to inter-channel coupling among the different channels in an OAM multiplexed link; (iii) the interference between the reflected and direct beams can induce intra-channel coupling between the received signals from the reflected and direct beams; and (iv) beams with a higher OAM order (e.g., from ± 1 to ± 5) or a lower carrier frequency (e.g., from 0.1 to 1 THz) experience larger intra- and inter-channel coupling. The intra- and inter-channel coupling in an OAM-multiplexed THz link can degrade the signal-to-noise ratio (SNR) and induce SNR penalty when compared to a single-channel system.

## Introduction

Structured electromagnetic (EM) waves have gained much interest partially due to their unique spatial beam structures with tailored amplitude and phasefronts^1,2^. One type of structured EM wave is orbital-angular-momentum (OAM)^1,3,4^. A beam carrying OAM has a unique spatial structure, such that the amplitude has a ring-like doughnut profile and the phasefront “twists” in a helical fashion as it propagates. OAM beams are a subset of the complete Laguerre Gaussian (LG_*ℓ, p*_) modal basis set, which has two modal indices: (i) *ℓ* represents the number of 2π phase shifts in the azimuthal direction, which is the OAM order, and (ii) *p* + 1 represents the number of concentric intensity rings^3,4^.

OAM beams with different OAM orders are orthogonal to each other. This orthogonality between different OAM modes could enable the data-multiplexing technique of mode-division multiplexing (MDM), which is a subset of space-division multiplexing (SDM)^5,6^. In this approach, multiple independent data-carrying OAM beams can be efficiently multiplexed at the transmitter, spatially co-propagate through the same medium, and be demultiplexed at the receiver, all with little inherent crosstalk. Therefore, the data capacity can be multiplied by the number of multiplexed beams^5^. There has been much progress in using OAM multiplexing to potentially enhance the data capacity in electromagnetic and mechanical systems across different frequency ranges, including microwaves, millimeter waves, and acoustic waves^5–14^.

There has been an emerging area in terms of using the terahertz (THz) band for wireless communication links^15–24^. In addition, future THz communication systems could potentially benefit from various types of integration, including: (i) metasurfaces for manipulating the phase and amplitude of the transmitted THz fields^25–28^, (ii) topological waveguides for guiding the THz signal on chips^29^. As a frequency range between millimeter waves and optical waves, THz waves might leverage the influence of divergence and light-matter interaction on wireless communications: (a) Compared to millimeter waves, THz waves tend to have a larger spectrum for communications and lower beam divergence for longer-distance transmission^24^; and (b) compared to optical waves, THz waves tend to exhibit lower light-matter interaction and are less affected by deleterious conditions (*e.g.*, atmospheric turbulence, rain, and fog)^19,30^. Recently, there have been reports on the use of OAM multiplexing to increase the data capacity of a THz wireless communication link^31^.

For OAM-based THz MDM links, there are several technical challenges and limitations due to the divergence of the propagating OAM beams, including (a) the limited-size aperture induced truncation of the structured data-carrying beams at the receiver^32–35^; and (b) multipath effects from a reflecting surface induced intra-channel power coupling (e.g., power coupling among different paths in the same OAM data channel) and inter-channel power coupling (e.g., power coupling among different OAM data channels)^36–41^. In^36^, simulation and experimental results are presented for intra- and inter-channel coupling caused by the multipath effects for millimeter waves ~ 28 GHz. However, the limited size of the receiver aperture was not considered for long-distance transmission. Meanwhile, the modal coupling analysis was done with the OAM modal set, which is the 1-D subset of LG modes with the indices *p* = 0, but not the 2-D LG modal set. A 2-D modal spectrum analysis might help the understanding of the modal coupling induced by different effects. Therefore, it would be important to explore the modal power coupling (a) across the full two-dimensional (2-D) LG modal spectrum; (b) over a wide frequency range (e.g., from 0.1 to 1 THz); and (c) by separating the direct and multipath channel-power components for intra- and inter-channel interference. We note that previous investigations of multipath effects for millimeter waves were done with only the OAM (*p* = 0) subset, but not the full LG set^36–41^.

In this paper, we simulate and analyze the limited-size receiver aperture and multipath effects in a THz wireless communication link using multiplexed OAM modes. We analyze the 2-D LG spectrum (e.g., *ℓ* from − 10 to + 10 and *p* from 0 to 20) created by the transmission of an OAM mode with *ℓ* =  + 3, *p* = 0 at a carrier frequency of 300 GHz when affected by the limited-size receiver aperture and multipath effects. Furthermore, we discuss the intra- and inter-channel coupling in different OAM links by varying the transmitted mode order (*e.g.*, *ℓ* = 0, + 1, + 3, *p* = 0), frequency (e.g., 0.1–1 THz), and multiplexed OAM links with the transmitted OAM order including *ℓ* =  ± 1, ± 3, and ± 5. The simulation results show that (i) a limited-size receiver aperture can induce power loss and modal power coupling mainly to LG modes with the same *ℓ* but *p* > 0 for directly propagated OAM beams; (ii) the multipath effect can induce modal power coupling across multiple two-2-D LG modes, which leads to inter-channel coupling among the different channels in an OAM multiplexed link; (iii) the interference between the reflected and direct beam can induce intra-channel coupling between the received signals from the reflected and direct beams; and (iv) beams with higher OAM order (e.g., from ± 1 to ± 5) or a lower carrier frequency (e.g., from 0.1 to 1 THz) experience larger intra- and inter-channel coupling.

## Results

### Concept and simulation model

**Figure 1:**
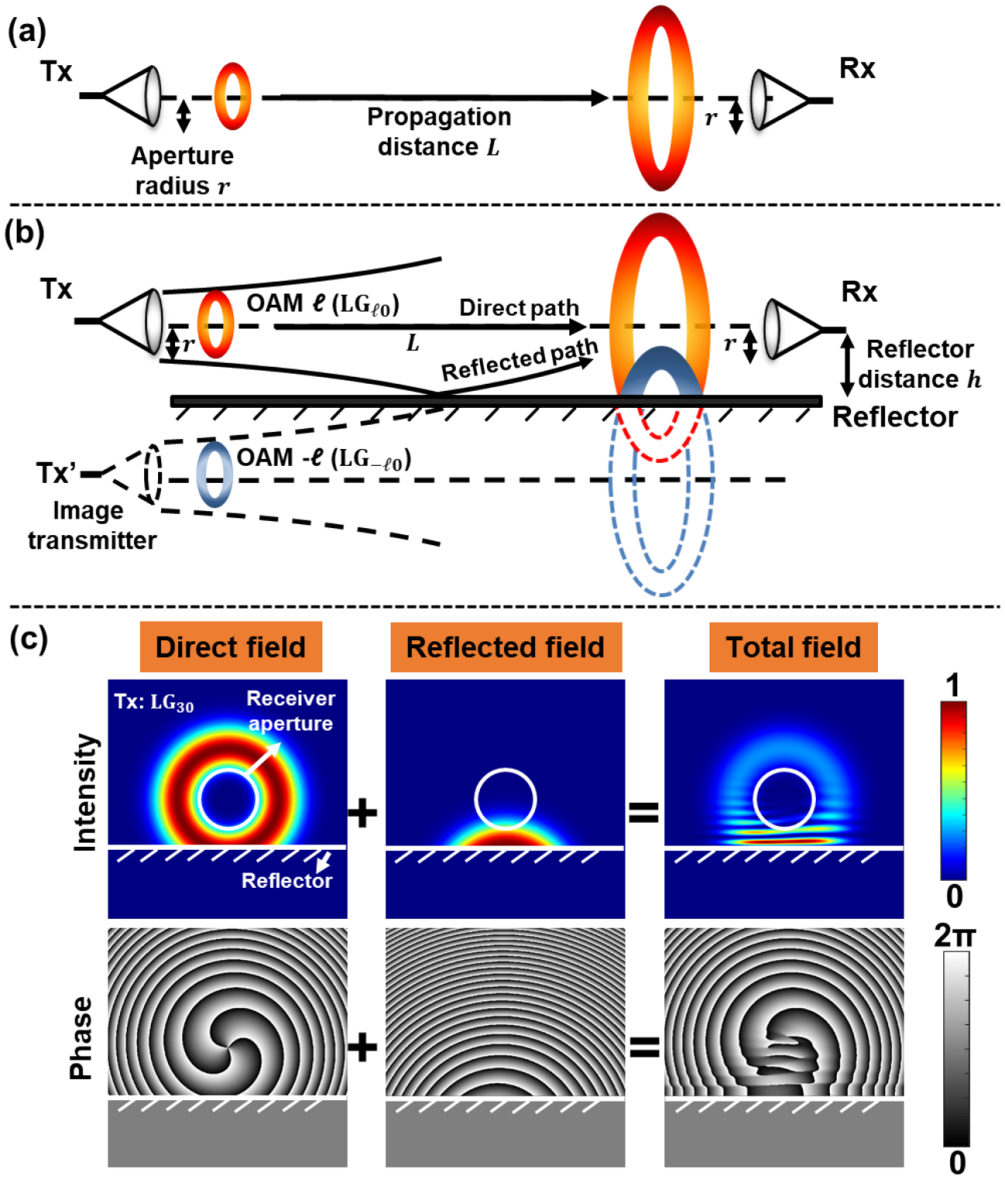
Schematic of limited-size receiver aperture and multipath effects in a THz OAM link. (**a**) Limited-size receiver aperture effect: Transmitted OAM beam propagating in free space can be circularly truncated by the receiver aperture. (**b**) Multipath effect: With a reflector placed in parallel with the propagation direction, the transmitted OAM beam can get reflected by the reflector. Subsequently, the fields from the direct and reflected path are collected by the receiver aperture. (**c**) Intensity and phase profiles of the direct, reflected, and total fields in the multipath effect when OAM + 3 is transmitted. The reflector is assumed to be perfect metal and can purely reflect the beam. Tx: transmitter; Tx’: image transmitter; Rx: receiver.

Figure 1 illustrates a schematic of the limited-size receiver aperture and multipath effects on THz communication links using OAM beams with *p* = 0 (i.e., ${\mathrm{LG}}_{ \ell0}$ beams). Figure 1a shows the limited-size receiver aperture effect. As shown in Fig. 1a, an OAM beam of order *ℓ* is transmitted from a transmitter (Tx) with an aperture radius of $r$. Due to the divergence of the beam, after propagating over a distance of *L*, the received beam experiences circular truncation by the limited-size receiver (Rx) aperture. Figure 1b shows the multipath effect on the OAM beam caused by a reflection from a reflector placed in parallel with the beam’s propagation direction at a distance of $h$ from the beam center. In this case, a part of the OAM beam will be reflected by the reflector (e.g., reflected field), while the rest of the beam propagates directly towards the Rx (e.g., direct field). The total field, which is the coherent sum of the direct and reflected fields, is cropped by the receiver aperture. The intensity and phase profiles of the direct, reflected, and total field (OAM + 3 is transmitted as an example) are shown in Fig. 1c. We assume an ideal reflector purely reflects the beam (planar reflection of 100% reflectivity) and the Rx is aligned with the center of the Tx aperture. The electrical field of the directly propagated beam is laterally truncated and reflected by the reflector during its propagation. The electrical field of the reflected beam can be regarded as a laterally truncated OAM field with an opposite OAM order -*ℓ* transmitted from an image transmitter (Tx’) placed at a distance of $2h$ from the original Tx^36^. The direct and reflected fields interfere, resulting in interference fringes in the total field. If the Rx has a limited aperture size, the received beam profile along the radial direction is also truncated. The 2-D LG modal spectrum of the received field is subsequently distorted.

**Figure 2:**
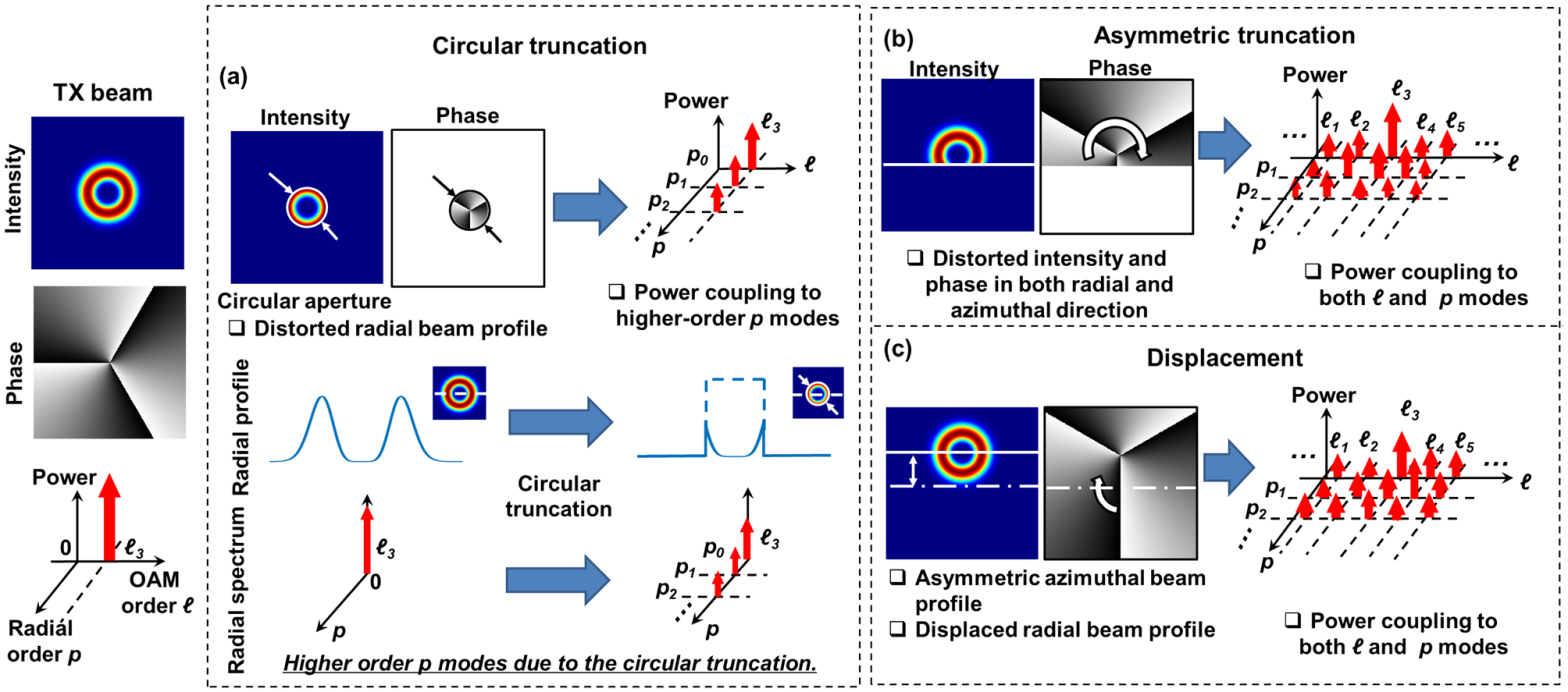
Concept of modal power coupling across the 2-D LG spectrum induced by different distortion effects on the received OAM beam. (**a**) Circular truncation effect induced by the limited-size receiver aperture. (**b**) Asymmetric truncation and (**c**) displacement effects induced by the multipath effect. OAM + 3 is transmitted as an example.

The limited-size receiver aperture and multipath effects induce power coupling across multiple LG modes, which can be interpreted as the consequence of a combination of several general distortion effects such as circular truncation, asymmetric truncation, and displacement. As shown in Fig. 2a, a circular limited-size aperture truncates the ring-shaped intensity profile of the received OAM beam along the radial direction. Since the aperture is circular and aligned with the transmitted beam, the beam profile is still azimuthally symmetric and the phase change in the azimuthal direction remains undistorted. In this case, circular truncation induces power loss to the truncated beam as well as modal power coupling mainly to LG modes with the same *ℓ* value (i.e., azimuthal modal indices) but higher *p* values (i.e., radial modal indices, *p* > 0)^32,34^. Different from the receiver aperture effects, the multipath effect generally consists of (i) an asymmetric lateral truncation of the direct field, and (ii) both asymmetric lateral truncation and displacement of the reflected field. Figure 2b shows the asymmetric truncation on the OAM beam. For the asymmetric truncation, the intensity and phase profiles of the OAM beam are affected in both the radial and azimuthal directions, and the beam profile is no longer azimuthally symmetric. For the displacement (Fig. 2c), the ring-shaped intensity and helical phase profiles remain relatively unchanged for the directly transmitted beams. However, the displacement effect does induce a translation between the center of the receiver and the transmitted beam. As a result, there is an intensity and phase mismatch between the ideal receiving field and the displaced field. For a perfectly aligned OAM beam at the receiver: (i) the intensity and phase profiles are circularly symmetric in relation to the receiver center, and (ii) the number of 2π phase changes along the receiver center indicates the OAM order. While in both cases, the intensity profiles are distorted, and there is a phase mismatch at the beam center. Consequently, both the asymmetric truncation and displacement effects induce power coupling to both the neighboring *ℓ* and *p* modes.

For an OAM-multiplexed link, the modal coupling caused by the limited-size receiver aperture and multipath effects induces both intra- and inter-channel coupling. The intra-channel coupling is defined as the power coupling from one OAM channel on the reflected path to the same OAM channel on the direct path. Consequently, the intra-channel coupling causes (i) power variation due to the constructive and destructive interference between the signals from the direct and reflected paths^42,43^ and (ii) inter-symbol interference due to the differences in the propagation distance between the direct and reflected paths^44^. In an OAM-multiplexed link, the inter-channel coupling is the power coupling among different data channels each carried by an OAM beam with a different transmitted OAM order. Such power coupling mainly comes from asymmetric truncation- and displacement-induced power coupling across the 2-D LG spectrum in the reflected field. Power coupling is further influenced by the limited-size aperture. This inter-channel coupling induces crosstalk in the OAM channels and results in degradation of the system performance.

### LG spectral analysis of the receiver aperture and multipath effects

We first simulate and analyze the modal power coupling induced by the receiver aperture and multipath effects by calculating the 2-D LG spatial spectra of the received THz OAM beams (see Methods for more simulation details). An LG spectrum with *ℓ* and *p* ranging from − 10 to 10 and from 0 to 20 is analyzed. As a proof of concept, an OAM + 3 beam with a beam waist ${w}_{0}$ of 5 cm and frequency *f* of 300 GHz is transmitted. The radii *r* of the transmitter and receiver aperture are both 10 cm when an OAM + 3 is transmitted (see Methods for more assumption details).

#### Limited-size receiver aperture effect

**Figure 3:**
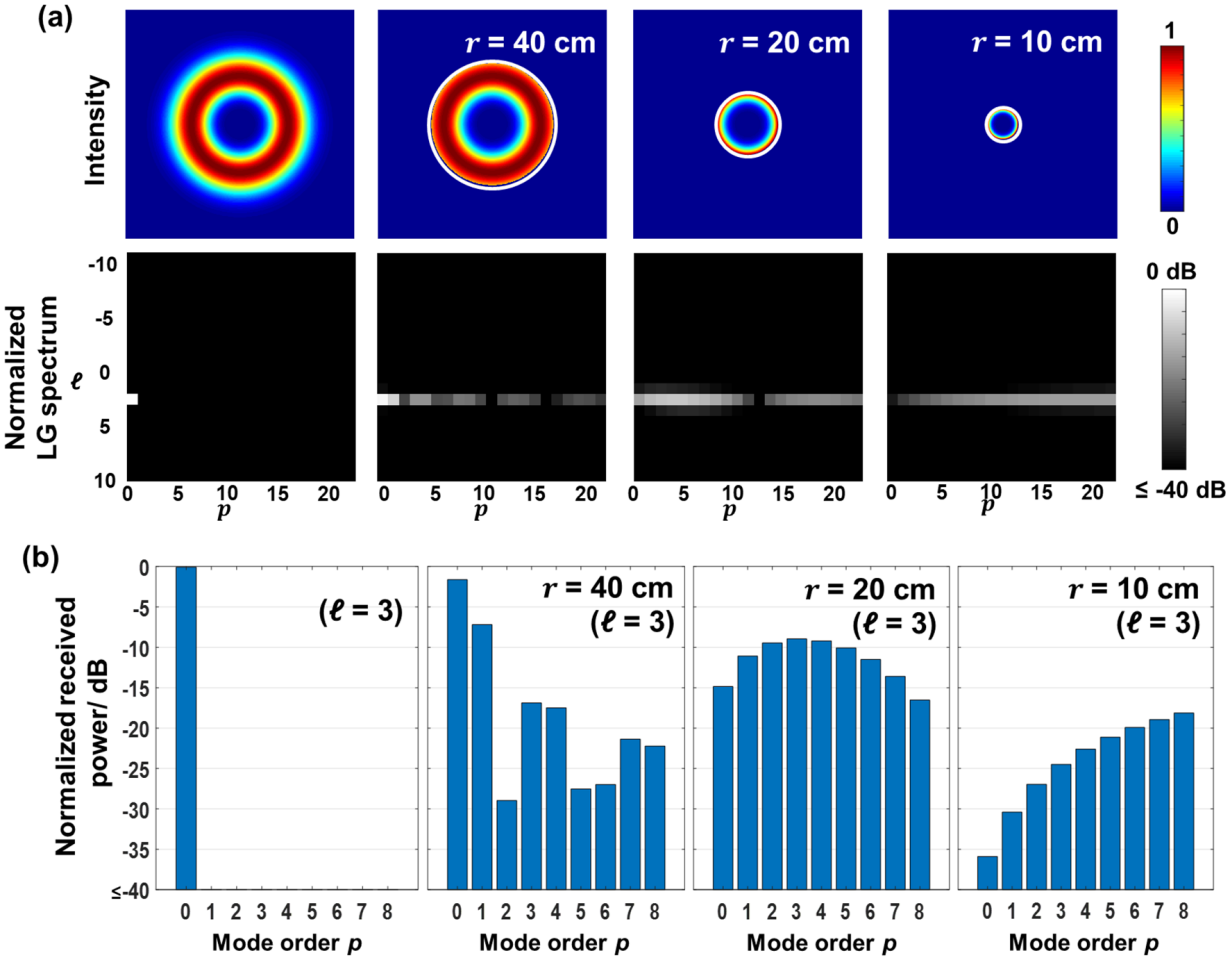
Simulated limited-size receiver aperture effect on directly propagated OAM beam. (**a**) Intensity profiles and 2-D LG spectra of an OAM + 3 beam received by a receiver with an aperture size large enough to capture the whole beam and a limited aperture size with a radius of 40, 20, and 10 cm. (**b**) LG power spectra with *p* from 0 to 8 and *ℓ* =  + 3 with a large enough aperture radius and an aperture radius of 40, 20, and 10 cm. The propagation distance *L* is 40 m.

Figure 3a shows the intensity profiles and LG spectra of an OAM + 3 beam that directly propagates in free space and is truncated by a limited-size receiver aperture. The propagation distance *L* is 40 m. When the aperture size is large enough to capture the whole beam, all modal power is contained in the ${\mathrm{LG}}_{30}$ mode. However, with the decrease in the receiver aperture radius from 40 to 10 cm, the received OAM beam is truncated by the aperture, causing power loss and power coupling mainly to neighboring *p* modes, while most of the received power remains in the LG modes with the same $\ell=+3$. This might be explained by that the circular aperture induces intensity and phase distortion along the radial direction but causes little degradation in the azimuthal spiral phase structure of the OAM beam^32,34^. Consequently, for an OAM link, the limited-size receiver aperture mainly induces power loss to the transmitted channel. We note that with 2-D LG modes transmitted, radial power coupling would also contribute to inter-channel coupling to other LG channels with the same *ℓ* but different *p* values. Figure 3b shows the LG spectra with *p* from 0 to 8 ($\ell=+3$) for different aperture radii. With a receiver aperture radius larger than 40 cm, the majority of the beam is not truncated by the receiver aperture, and the modal components remain on the *p* = 0 mode. With a further decrease in the receiver aperture radius, a smaller aperture size induces a stronger distortion and causes modal power coupling to higher-order *p* modes. The modal power dips at different *p* values. This phenomenon can be explained by the role of the circular aperture as a rectangular sampling function in the radial direction. The Fourier transform of the rectangular window samples the modal power across the radial (*p*) spectrum^32–34^. We note that, in our simulation, a limited number of *p* modes are used for decomposition, and the entire modal power distribution requires further exploration.

It should be noted that if the multipath effect is also considered, since the aperture radius *r* is assumed to always be smaller than the displacement *h* of the reflector from the axis, the received field of the direct path captured by the limited-size aperture is not influenced by the truncation due to the reflector. Thus, the LG spectrum of the direct field is the same as that of a directly propagated OAM beam*.*

#### Multipath effect

**Figure 4:**
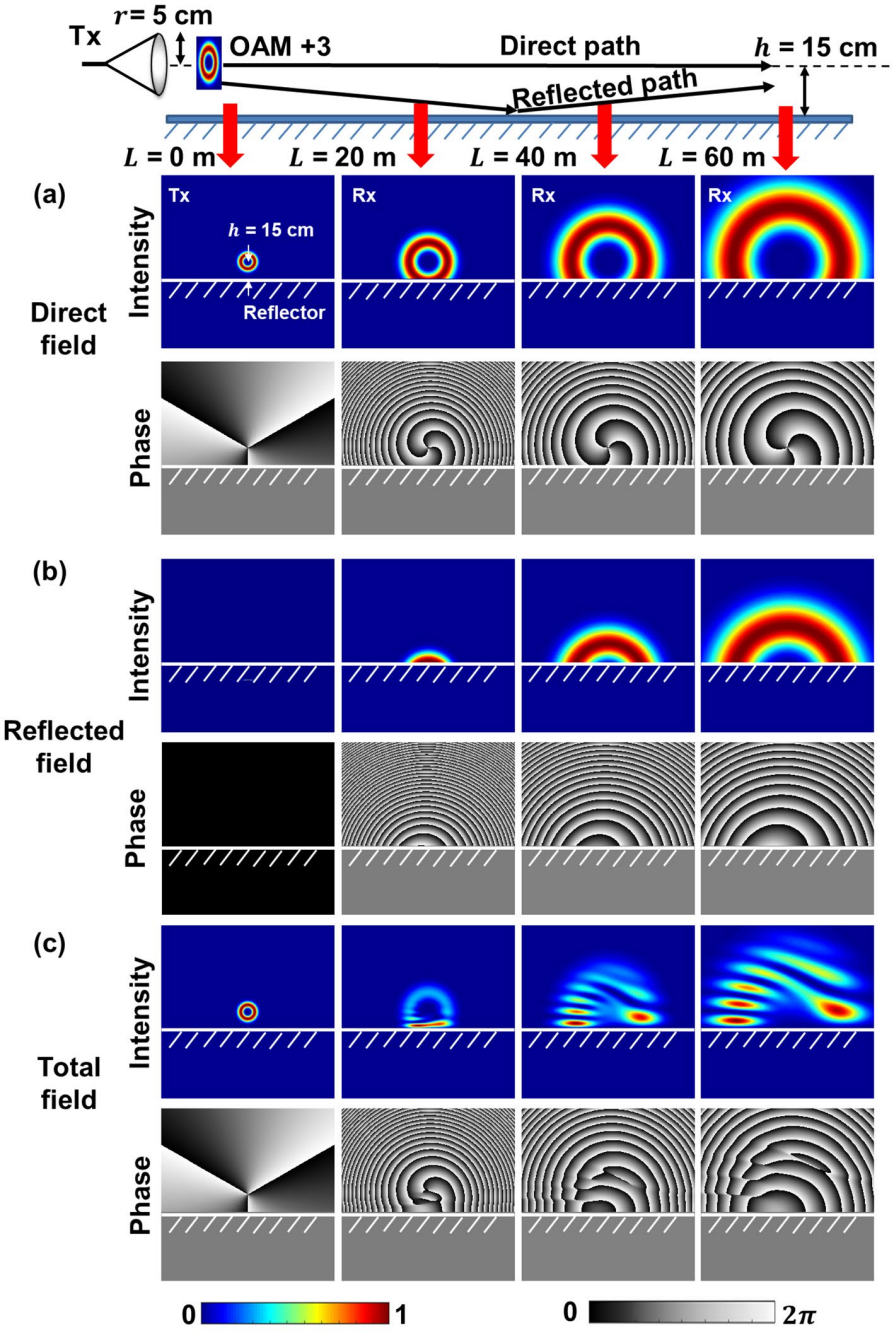
Simulated intensity and phase profiles of (**a**) the direct field, (**b**) the reflected field, and (c) the total field at propagation distances *L* of 0, 20, 40, and 60 m.

Next, we simulate the multipath effect as a combination of lateral truncation and displacement on the fields of pure OAM beams. Specifically, we simulate (i) the direct, reflected, and total field profiles; (ii) the LG modal coupling of the reflected field; and (iii) the LG modal coupling of the total field, which is a coherent sum of the direct and reflected field. As an example, Fig. 4 shows the simulated intensity and phase profiles of an OAM beam influenced by the multipath effect at different propagation distances up to 60 m. The reflector distance *h* is set to be 15 cm. OAM + 3 is transmitted in this example. The beam waist ${w}_{0}$ is set to be 5 cm. We assume that an aperture large enough to capture both the direct and reflected beam profiles is used at Rx. Figure 4a shows the intensity and phase profiles of the electrical field created by the direct path. With the increase in the propagation distance, a larger portion of the transmitted beam is laterally truncated by the reflector. Figure 4b shows the profiles of the field from the reflected path which is equivalent to that of a truncated OAM -3 beam with a lateral displacement to the direct path. As shown in Fig. 4c, since the direct and reflected fields are coherent to each other, the total received fields represent the interference among the fields. Because the OAM order of the direct field is opposite to that of the reflected field, and there is an offset between the beam center of the direct and reflected fields, the interference has a fork pattern in the intensity profile. With the increase in propagation distance, due to the diffraction of the beam, the beam size of the transmitted beam increases^31,45^, and larger portions of the direct field tend to be reflected and contribute to the reflected field, leading to a stronger distortion in the beam profile of the total field^36^. We note that if the receiver aperture is not considered, when the distance approaches the infinity, the beam size of the transmitted beam grows with the distance. Since the transmitter is above the reflector, due to this geometry, at most nearly half of the beam will tend to be reflected, and the other half of the beam will remain on the direct path^36^. As a result, the total field could become the superposition of two half OAM beams with the opposite OAM order and a certain offset. However, in practice, the link distance could be limited due to the diverged beam size and the receiver aperture size.

**Figure 5:**
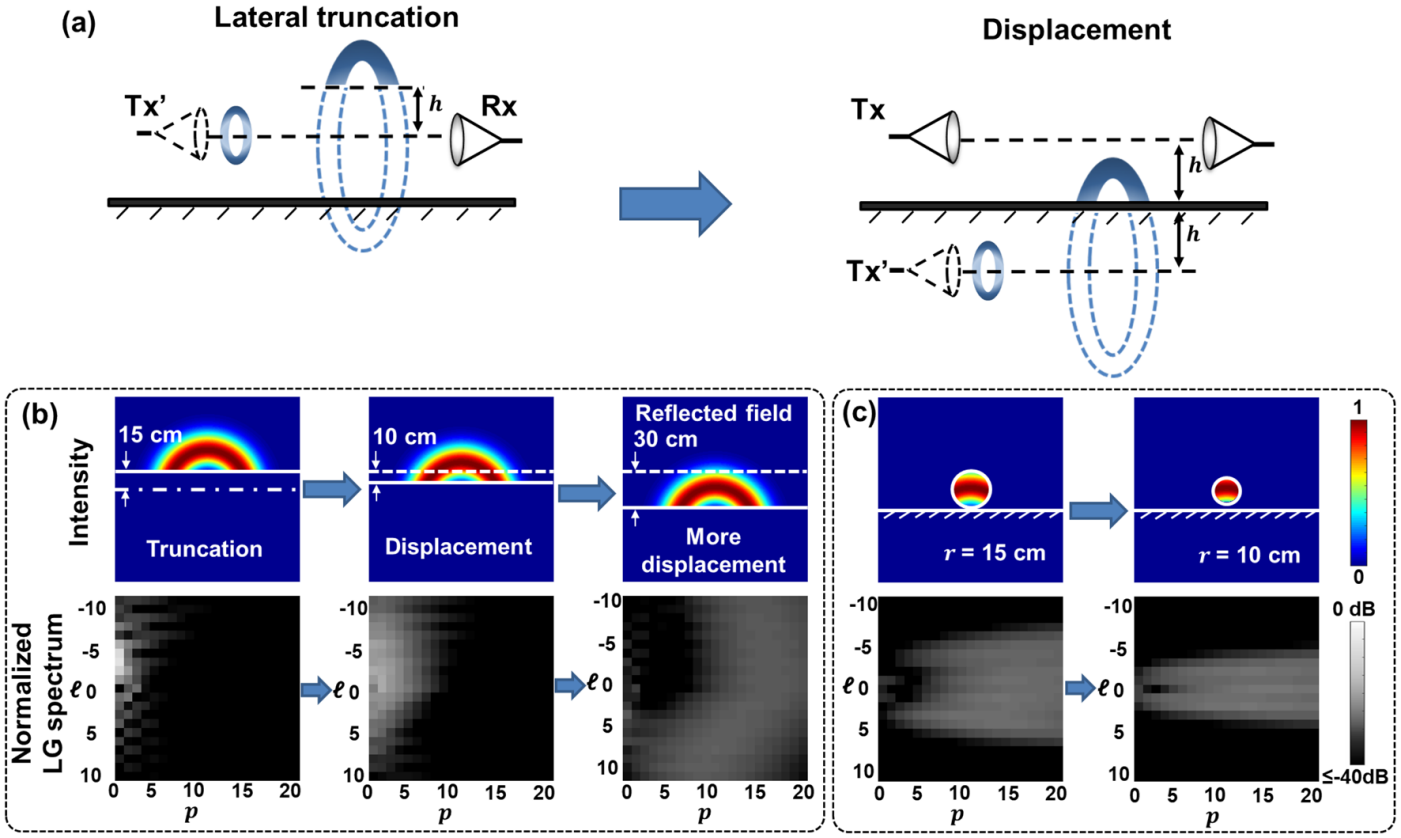
Simulated multipath and limited-size receiver aperture effects on the reflected field. (**a**) Intensity profiles and 2-D LG spectra of the reflected field affected by the multipath effect, which consists of lateral truncation and displacement. The dash-dotted line indicates the center of the direct field; the dashed line indicates the edge of the truncated beam without displacement. (**b**) Concept of the simulated reflected field. (**c**) Intensity profiles and 2-D LG spectra of the reflected field with a limited-size receiver aperture. The propagation distance *L* is 40 m, and the reflector distance *h* is 15 cm.

Figure 5 presents the LG modal power coupling of the reflected field induced by the multipath effect with and without consideration of the limited-size receiver aperture. As shown in Fig. 5a, the reflected field with reflector distance *h* is simulated by the transformation as follows: (i) a pure OAM -*ℓ* beam is truncated with the distance between the edge and the beam center of *h*; and (ii) subsequently, the laterally truncated beam is translated with a lateral displacement of 2 h. The calculated 2-D LG spectra in Fig. 5b show that (i) the lateral truncation effect leads to power coupling to both neighboring *ℓ* and *p* modes due to the distortion along both the azimuthal and radial directions; and (ii) the increase in displacement from 0 to 2 h (30 cm) might induce further power coupling to higher *ℓ* modes and *p* modes as the mismatch between the distorted field and the ideal LG mode along both the azimuthal and radial directions becomes stronger^46^. Furthermore, the LG spectra of the simulated field from the reflected path under the effect of the limited-size aperture are shown in Fig. 5c. With the decrease in the receiver aperture radius from 15 to 10 cm, the power coupling to higher *ℓ* modes decreases. This might be due to that the higher-order *ℓ* modal components generally have a larger ring-shaped intensity profile, and there is little power at the beam center that can be detected by the aperture.

**Figure 6:**
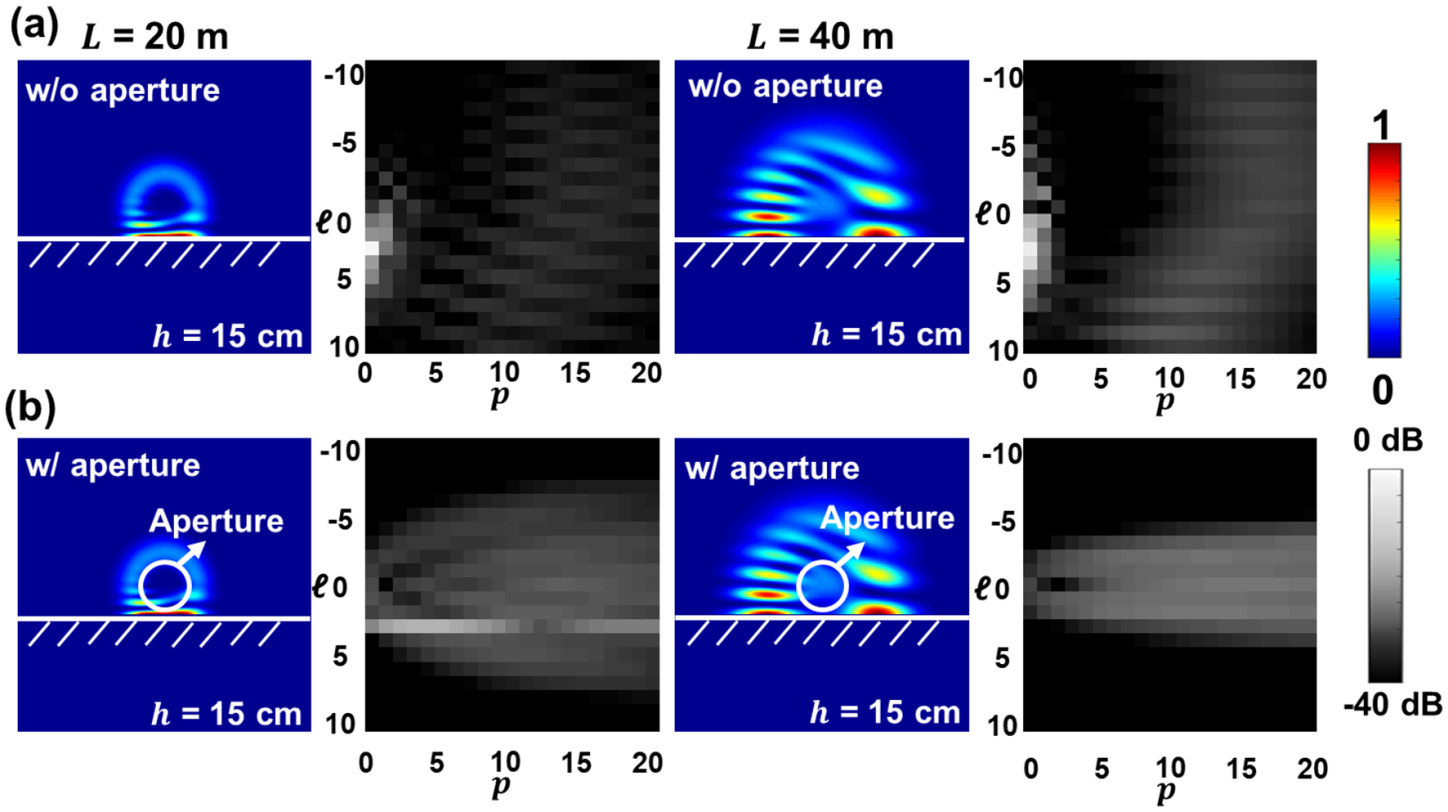
Simulated multipath and receiver aperture size effects on the total field. Intensity profiles and 2-D LG spectra of the total field at propagation distances *L* of 20 and 40 m (**a**) without and (**b**) with the limited-size receiver aperture. The reflector distance *h* is 15 cm.

We also analyze the modal power coupling of the total field (i.e., a superposition of the field from the direct path and reflected path) at different propagation distances *L*. Figure 6a shows the intensity profiles and 2-D LG spectra of the total field without considering the limited-size receiver aperture at different *L*. The simulation results indicate that an increased propagation distance *L* from 20 to 40 m induces more power coupling to higher *ℓ* and *p* modes. This might be caused by the fact that a larger portion of the beam is truncated and reflected by a reflector with a longer propagation distance due to the beam divergence. Furthermore, Fig. 6b presents the LG spectra under the effect of a limited-size receiver aperture. At *L* = 20 m, a larger portion of the modal components remains on the *ℓ* =  + 3 mode, and this might be due to that only little power is reflected and most of the power remains on the direct path. With the propagation distance increased to *L* = 40 m, less modal power remains on *ℓ* =  + 3 modes. Meanwhile, compared with the spectrum at *L* = 20 m, the effect of the limited-size aperture accompanied by the beam divergence induces lower power coupling to higher *ℓ* modes. This might be explained by that with the increase in the propagation distance, higher-order *ℓ* modal components have a larger divergence effect and tend to be filtered out by the limited-sized aperture.

In this section, we analyze the modal power coupling across the 2-D LG spectra regarding the receiver aperture and multipath effects. Due to the diffraction of free space beams, the beam size will increase with the propagation of the beam. Consequently, a limited-size receiver aperture will truncate the received fields. Since the OAM beams are circularly symmetric, a circular truncation caused by the aperture induces modal power coupling mainly to radial *p* modes instead of azimuthal *ℓ* modes. However, for the multipath effect, since the received fields would be distorted in both azimuthal and radial directions, modal coupling to both *ℓ* and *p* modes could be expected.

### System performance of multipath and receiver aperture affected OAM multiplexing systems

We further analyze the received power, intra-channel coupling, and inter-channel coupling of a THz OAM link under different multipath and receiver aperture conditions by simulation. In our simulation, the received power is normalized to the transmitted power. Subsequently, the unit of the received power is decibel (dB). In this case, the received power also indicates the power loss of the link for each channel.

#### Propagation distance

**Figure 7:**
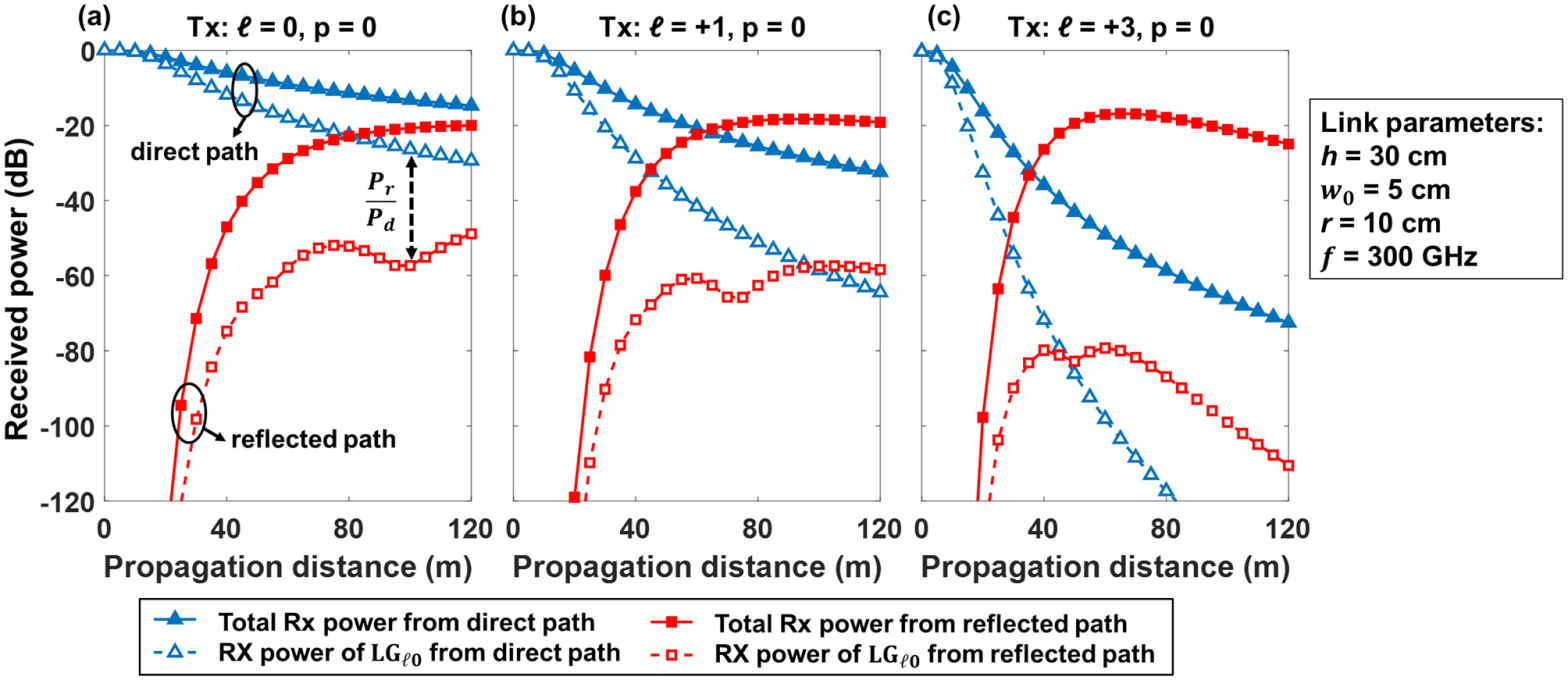
Simulated received power for (**a**) the Gaussian (*ℓ* = 0), (**b**) OAM + 1, and (**c**) OAM + 3 beams at different propagation distances. The double arrow line indicates the reflected-to-direct power ratio.

We first analyze the received power from the direct and reflected paths for different OAM orders as a function of propagation distance in consideration of the multipath and limited-size receiver aperture effects. Both the total received power, which consists of all *ℓ* and *p* modal components, and the received power of only the desired OAM mode (${\mathrm{LG}}_{ \ell,0}$) are simulated. In Fig. 7, for the transmitted OAM beam of *ℓ* = 0, + 1, + 3, the total power collected by the receiver aperture from the direct path decreases by ~ 6, ~ 14, and ~ 35 dB, respectively, with the propagation distance increasing from 0 to 40 m. The higher power loss for a higher-order OAM beam is mainly due to the larger beam divergence of higher-order OAM beams^45^. At *L* = 40 m, there is an extra power loss of ~ 5, ~ 14, ~ 33 dB for the received power of the ${\mathrm{LG}}_{00}$, ${\mathrm{LG}}_{10}$, and ${\mathrm{LG}}_{30}$ modes from the direct path, respectively, compared with the total power from the direct path. This might be because the limited aperture size induces power coupling from the transmitted OAM modes to mainly the same *ℓ* but higher-order *p* (*p* > 0) modes as discussed in the previous section. Similarly, for the reflected path, the larger divergence of the higher-order OAM beams allows for a larger portion of the beam to be reflected. As a result, when the OAM + 3 is transmitted, the total received power of all the modal components from the reflected path at *L* = 40 m is ~ 14 dB, which is ~ 4 dB higher than the scenarios where the Gaussian (*ℓ* = 0) and OAM + 1 beams are transmitted, respectively. Moreover, the ratio between the received power of ${\mathrm{LG}}_{ \ell0}$ from the reflected path (*P*_*r*_) and that from the direct path (*P*_*d*_) on the transmitted OAM order *ℓ*, which is defined as the reflected-to-direct power ratio (*P*_*r*_/*P*_*d*_) indicates intra-channel coupling. As the propagation distance increases from 0 to 60 m, the received power from the direct path decreases, while the reflected power increases. As a result, the reflected-to-direct power ratio increases in this range. With a further increase in the distance from 60 to 120 m, the received power from the direct path still decreases. However, the received power from the reflected path saturates, and may decrease. This could be because the diverged reflected beam tends to cover the whole receiver aperture after a certain distance. Furthermore, the power density captured by the receiver aperture decreases with the increase in the beam size. In this case, since the received power from the direct path decreases faster, as shown in Fig. 7, the reflected-to-direct power ratio still generally increases with the increase of propagation distance.

**Figure 8:**
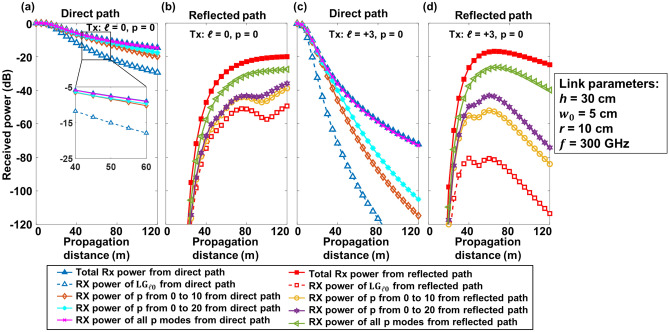
Simulated total received power and received power of *p* = 0, *p* from 0 to 10, *p* from 0 to 20, and all *p* modes from (**a**) the direct path, (**b**) the reflected path of the Gaussian (*ℓ* = 0) beam, and (**c**) the direct path, (**d**) the reflected path of OAM + 3 beam at different propagation distances. The inset in (**a**) shows a zoomed figure of the receiver power with a propagation distance from 40 to 60 m.

As discussed in the previous section, the multipath and limited-size receiver aperture effects induce power coupling to higher-order *p* modes. Thus collecting the power from the non-zero *p* modes received by the receiver aperture might help reduce the power loss in a THz OAM link^35^. Figure 8 shows the received power of (i) *p* = 0, (ii) *p* from 0 to 10, (iii) *p* from 0 to 20, and (iv) all *p* modes for a transmitted *ℓ* value from the direct and reflected paths. For the direct path, as shown in Fig. 8a, c, with an increasing number of *p* modes at *L* = 40 m, the received powers increase by ~ 5 and ~ 44 dB for the Gaussian and OAM + 3 beams, respectively. Since the circular aperture would barely distort the spiral phase pattern of the OAM beams in the direct path, there is little power coupling to the LG modes with other *ℓ* values. Therefore, the received power of all *p* modes is close to the total received power from the direct path. It should be also noted that, by collecting *p* modes from *p* = 0 to 20, the received direct path power of the Gaussian beam is almost equal to the received of all *p* modes, while that of the OAM + 3 beam is 10 dB less than the received of all *p* modes. This could be because the higher-order OAM beam has a larger beam size, thus the circular truncation would induce a greater portion of the modal power to be coupled to higher-order *p* modes. For the reflected path, as shown in Fig. 8b, d, the received power increases by ~ 17 and ~ 45 dB when collecting all *p* modes for the Gaussian and OAM + 3 beams, respectively. However, there is a ~ 10 dB power difference compared to the total received power from the reflected path for both the Gaussian and OAM + 3 beams. This might be due to power coupling from the transmitted mode to the other *ℓ* modes in the reflected path.

#### Frequency

**Figure 9:**
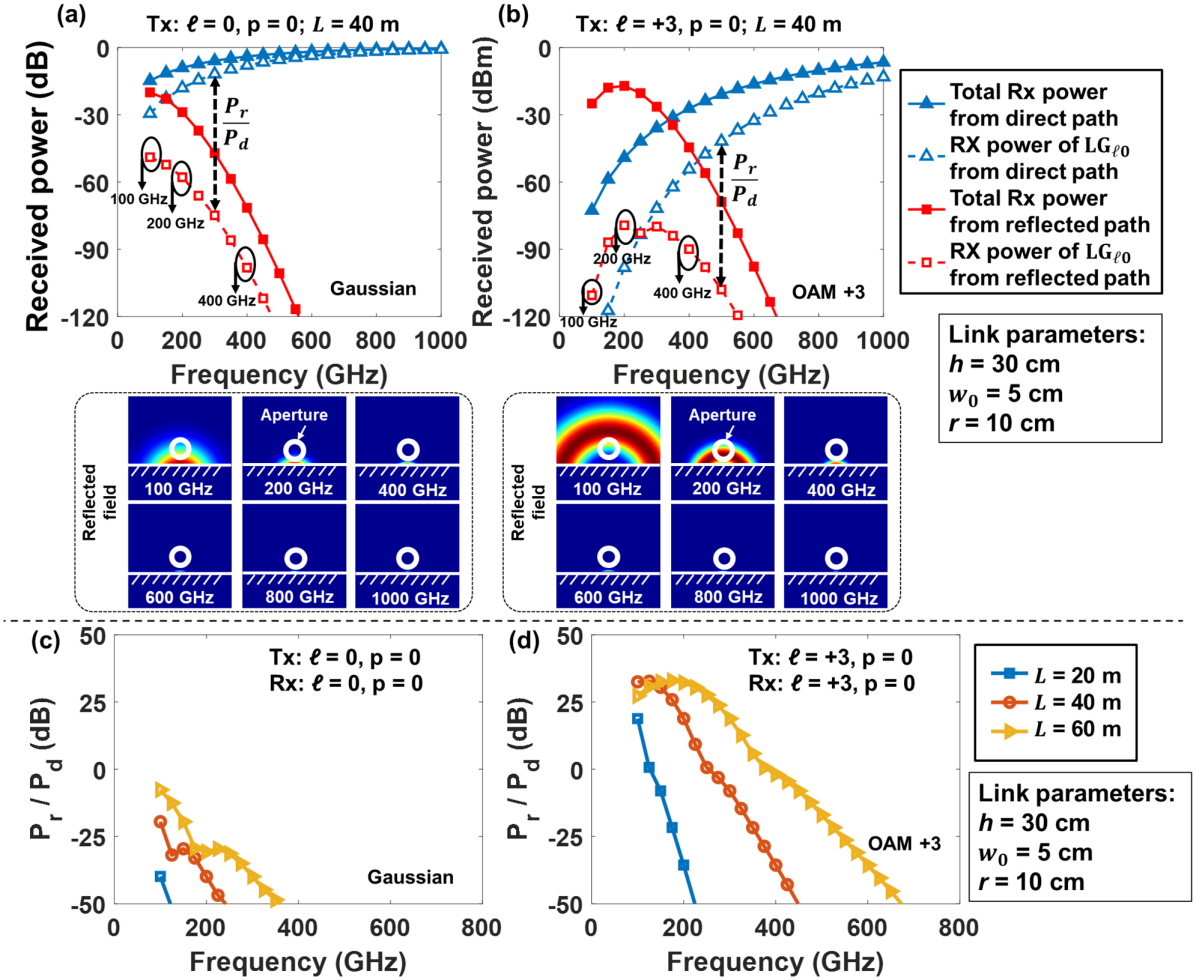
Simulated received power with (**a**) the Gaussian (*ℓ* = 0) and (**b**) OAM + 3 beams transmitted. Simulated reflected-to-direct power ratio with (**c**) the Gaussian and (**d**) OAM + 3 beams transmitted. For (**a**) and (**b**), the propagation distance *L* is 40 m. Inset figures show the reflected fields at the frequencies from 100 to 1000 GHz for transmitted (**a**) Gaussian and (**b**) OAM + 3 beams.

Moreover, we explore the multipath and receiver aperture effects on the received power and intra-channel coupling at different frequencies. As shown in Fig. 9a, b, we simulate the received power for the Gaussian and OAM + 3 beams at frequencies from 100 to 1000 GHz with *L* = 40 m. To help explain the trend of the received power in this specific simulation case, the insets of Fig. 9a, b show examples of the intensity profiles of reflected fields for Gaussian and OAM + 3 beams at frequencies from 100 to 1000 GHz. For the Gaussian and OAM + 3 beams, the received power of the corresponding *p* = 0 mode from the direct path increases by ~ 28 and ~ 110 dB, respectively, with an increase in frequency from 100 to 1000 GHz. This might be because the coupling power of the direct field mainly depends on the beam divergence, and a higher frequency or lower OAM mode tends to have less divergence and thus a larger portion of the beam is detected without truncation. For the reflected path, the received power of the Gaussian beam decreases with a higher frequency. However, for the OAM + 3, the received reflected power increases when *f* increases from 100 to 200 GHz and subsequently decreases when *f* increases from 200 to 1000 GHz. This phenomenon could be due to the ring-shaped intensity profile of the OAM beams. As shown in the insets intensity figures, for Gaussian beams, the larger divergence for lower frequencies causes a larger portion of the beam to be reflected and received by the aperture. However, OAM beams have a ring-shaped profile and a low-power region at the center of the beams. With the decrease in the frequency (e.g., from 400 to 200 GHz), a larger portion of the beam is reflected, and the overlapping between the receiver aperture and the reflected OAM beam first increases. Consequently, the reflected received power increases. When the frequency further decreases (e.g., from 200 to 100 GHz), the beam becomes larger, and the receiver aperture falls inside the ring profile. As a result, the overlapping between the receiver aperture and the reflected OAM beam decreases, and the reflected power decreases. In this particular simulation case, at a frequency of around 200 GHz, the larger overlapping area induces a peak in the power received from the reflected path. For different system parameters, the frequency of the peak-reflected power could change.

As shown in Fig. 9c, d, the reflected-to-direct power ratio decreases when (i) the frequency increases; (ii) the OAM order decreases; and (iii) the propagation distance decreases. This indicates that the reflected-to-direct power ratio mainly depends on the divergence effect, which is related to the frequency, mode order, and propagation distance.

#### Inter-channel coupling in an OAM multiplexing link

**Figure 10:**
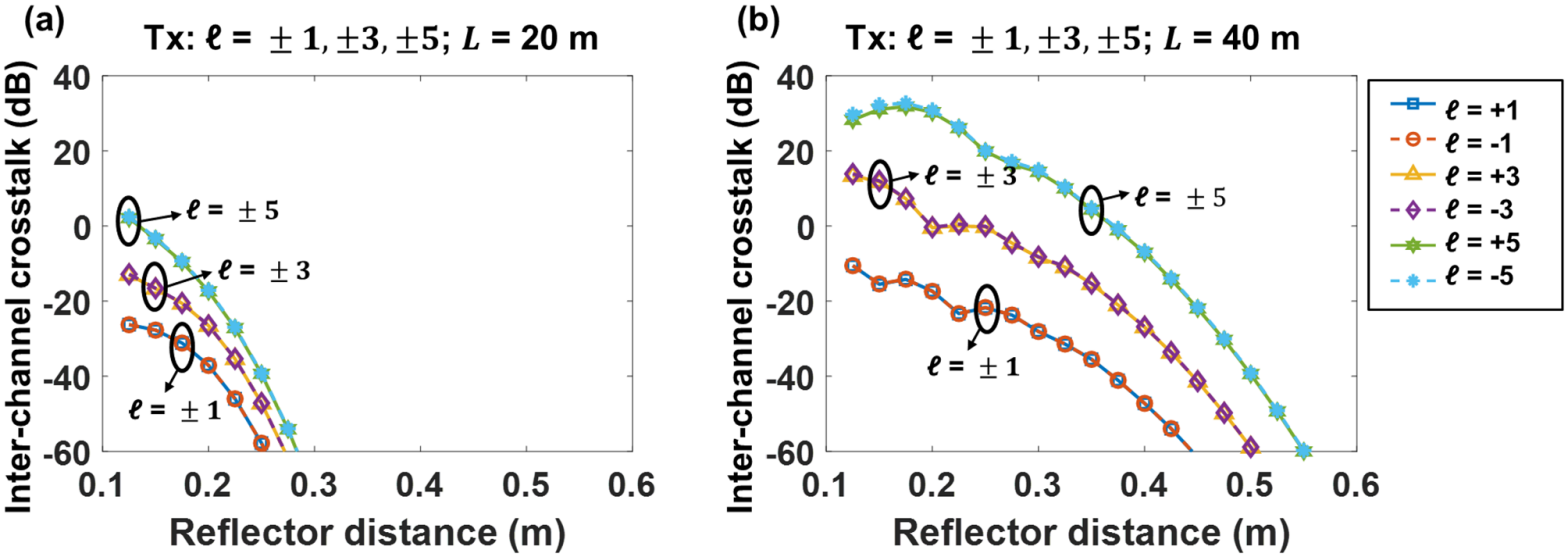
Simulated inter-channel crosstalk with OAM ± 1, ± 3, and ± 5 transmitted at propagation distances *L* of (**a**) 20 m and (**b**) 40 m. The beam waist *w*_*0*_ is 5 cm and the aperture radius *r* is 12.25 cm.

The modal coupling to neighboring *ℓ* modes caused by the multipath and receiver aperture effects induces inter-channel coupling if multiple OAM beams are simultaneously transmitted in a THz communication link. Here, we define the inter-channel crosstalk of the OAM $\ell$ channel as the ratio of the power coupled from other undesired OAM channels (transmitted OAM order different from $\ell$) to the signal power of the transmitted OAM $\ell$ channel. As a proof of concept, we analyze the inter-channel crosstalk in a THz link with 6 OAM beams multiplexed (OAM order *ℓ* =  ± 1, ± 3, and ± 5). The simulated radii of the transmitter and receiver apertures are set to 12.25 cm to cover the ring-shaped beam profile of transmitted beams with the highest OAM order *ℓ* =  ± 5. As shown in Fig. 10, we investigate the inter-channel crosstalk for different OAM channels varying the reflector distance *h*, which is the distance between the beam propagation axis and the parallelly placed reflector, from 0.125 to 0.6 m. With an increase in reflector distance, inter-channel crosstalk decreases. This can be explained by that, as *h* increases, it requires a larger divergence for the beam to reach the reflector, and a smaller portion of the beam is reflected. Furthermore, the inter-channel crosstalk of channels with OAM order *ℓ* =  ± 5 is ~ 20 dB larger than that of channels with OAM order *ℓ* =  ± 1 at *L* = 20 m. This might be because (i) a lower-order OAM beam tends to have a smaller beam size, and thus higher signal power is received from the direct path, and (ii) for different OAM channels, the power leakage from all the other OAM channels is similar^36^. Consequently, for an OAM channel with a lower transmitted OAM order, higher signal power leads to lower inter-channel crosstalk. Moreover, with the transmission distance increasing from 20 to 40 m, the inter-channel crosstalk for OAM ± 3 increases from ~ − 20 to ~ 0 dB with the reflector distance h of 0.2 m. This could be because at a longer distance, a larger portion of the transmitted beams is reflected and induces channel crosstalk. The crosstalk performance is generally similar for OAM channels with the same |*ℓ*|, and this can be explained by the symmetrical nature of OAM beams with the same |*ℓ*|.

In our simulation, the link distance varies from 0 to 120 m, considering our specific system parameter configurations. In this distance range, both the receive aperture and the multipath effects are shown. We note that, with a longer distance, due to beam divergence, the received signal power is relatively low for signal reconstruction. The link distance for a THz link could potentially be extended by transmitting beams with a larger beam waist and carrier frequency to reduce divergence.

In this section, we simulate the received power, intra- and inter-channel coupling for an OAM-based THz link considering both receiver aperture and multipath effects. The resulting degradation is generally induced by the power loss and modal power coupling caused by the reflection and truncation of the received beam, which is dependent on (i) the beam parameters (e.g., OAM order, frequency, and beam waist); and (ii) the link system parameters (e.g., the propagation distance, reflector distance, and aperture size).

## Summary and discussion

We simulate and explore multipath and receiver aperture effects in a THz wireless communication link using multiplexed OAM beams. Compared to previous reports^32–41^, our work uniquely explores the degradation effects caused by multipath and receiver aperture by analyzing the modal power coupling among 2-D LG modal spectra. The simulation results show that both effects induce power loss and power coupling in the 2-D LG spectra of THz OAM beams. Regarding the limited-size receiver aperture, it can induce modal power coupling mainly to LG modes with the same *ℓ* value but *p* > 0 values; and regarding the multipath effect, it can induce modal power coupling across multiple 2-D LG modes, which leads to both intra- and inter-channel coupling in an OAM multiplexed link. The resulting intra- and inter-channel crosstalk depends on (i) the OAM beam parameters, including OAM order, frequency, and beam waist; and (ii) the link system parameters, including the propagation distance, reflector distance, and aperture size. These results generally indicate possible power loss, 2-D modal coupling, and channel coupling regarding OAM-based THz links. The conclusions in this paper might potentially help the system design of THz OAM multiplexed wireless communication links with limited-size aperture and reflectors surrounded with the channel.

The results of our simulation primarily focus on the fundamental 2-D LG modal coupling induced by the limited-size aperture and multipath effects in an OAM multiplexing link. For application, more complicated system configurations need to be taken into consideration. A few factors are discussed as follows: (i) In our simulation, atmospheric absorption and environmental effects (e.g., atmospheric turbulence, fog, dust, rain, and snow) along the propagation are not considered. Time-variant environmental effects tend to distort the intensity and phase profiles of transmitted beams^47–49^. Meanwhile, they may affect the direct and reflected fields differently, thus influencing interference between the direct and reflected fields. Further simulation and experimental investigation may consider the combined impact of multipath effects and atmospheric absorption as well as considering different environmental distortions. (ii) With our system configuration, major results of the limited-size receiver aperture and multipath effects occur and are analyzed beyond the Rayleigh range (${z}_{R}=\pi {\omega }_{o}^{2}/\lambda \approx 7.8 \mathrm{m}$), which is regarded as the far field^50^. This is generally related to the system parameters and the divergence of the transmitted beam. Since the transmitted beam first diverges along the propagation direction, it is then reflected by the parallel reflector or truncated by the receiver aperture. The relatively small divergence in the near field leads to little receiver aperture and multipath effects in our simulated cases. However, if the divergence of the transmitted beam is increased (e.g., lower frequency or larger OAM order), there might also be receiver aperture and multipath effects occurring in the near field. (iii) Recent work reports an experimental demonstration of using 2-D LG modal sets for multiplexing without considering receiver aperture and multipath effects^51^. In this paper, we simulate a THz multiplexing system using OAM beams with p = 0, which is a subset of LG modes. The use of a 2-D set of modes (both *ℓ* and *p*) could potentially provide a larger set of channels and beams for such THz MDM systems. The future investigation could consider simulate and experimentally investigate the intra- and inter-channel coupling with the transmission of LG modes varying both *ℓ* and *p* indices. (iv) We assume the receiver aperture is always perfectly aligned with the incoming beams. However, a receiver pointing error could also induce angular and lateral misalignment between the transmitted beams and receiver aperture. Thus, the misalignment will also induce intensity and phase distortions, and degrade the system performance^45^. (v) In our simulation, we mainly investigate the channel power loss and intra- and inter-channel crosstalk for a THz OAM link. For a communication system, the SNR and bit error rate (BER) are also important parameters for analyzing the system performance. To calculate the SNR value, the signal power could be estimated using the channel power loss from this simulation. The noise power consists of different components, such as intra- and inter-channel crosstalk and noise from the receiver^50^. Our simulation provides an estimation of channel crosstalk, while receiver noise is not considered. Once the SNR of the link is estimated, the BER performance can be calculated as a function of the SNR regarding different signal modulation formats and orders (e.g., pulse-amplitude modulation, phase shift keying, and quadrature amplitude modulation)^50^.

Our investigation of receiver aperture and multipath effects is performed by simulation. Future exploration potentially considers experimentally investigating these effects. The challenges of experimental demonstration might include the following: (i) The inaccurate generation and detection of LG beams: The imperfection of generating OAM beams and modal spectrum analysis could degrade the accuracy of the results. (ii) The alignment of the transmitter, receiver, and reflector: The misalignment between these components would also distort the modal profile and induce additional power loss and channel crosstalk. (iii) Environmental distortions: Time-varying environmental effects including turbulence, fog, dust, rain, and snow, could dynamically distort the transmitted beams and induce power loss and crosstalk^47–49^.

## Methods

### Simulation details

In our simulation, we generate the received electrical fields by Matlab. Furthermore, the modal power components are calculated for the received beams to estimate power loss and channel crosstalk^36,47^.

We assume that the Tx and Rx in our simulated OAM link consists of a THz horn antenna and an OAM mode converter. THz antennas are usually designed with Gaussian profiles^15–24^. In this case, at the Tx, a THz Gaussian beam emitted from the transmitter antenna will propagate through the mode converter and be converted to a THz OAM beam. In addition, structured beams might be directly emitted from the transmitter without mode conversion from a Gaussian beam^52^. We note that, in practice, the imperfection of the Gaussian beam emitted by the Tx antennas and the impairment of OAM mode conversion would induce extra power loss and modal power coupling^31^. To isolate the effect of devices, we assume ideal Gaussian beams and perfect mode conversion are achieved by the system. At the Rx, on the contrary, the received OAM beam will be converted back to a Gaussian beam and captured by the Rx antenna. Due to the divergence of the beam, the beam size will increase with the distance at the Rx, and the phase profile will experience aberration^45^. Based on this assumption, we use the ideal OAM fields as our transmitted beams, and the specific OAM modal power components from the received beam are assumed to be the received power.

The electric fields of the OAM beams in this simulation are described by a subset of ${LG}_{ \ell,p}$ beams with a zero *p*-value, which is defined as^4^:

1 $$ \begin{gathered} LG_{\ell ,p} \left( {r,\theta ,z,\omega } \right) = \frac{{C_{\ell ,p}^{LG} }}{w\left( z \right)}\left( {\frac{r\sqrt 2 }{{w\left( z \right)}}} \right)^{\left| l \right|} \exp \left( { - \frac{{r^{2} }}{{w^{2} \left( z \right)}}} \right)L_{p}^{\left| \ell \right|} \left( {\frac{{2r^{2} }}{{w^{2} \left( z \right)}}} \right) \hfill \\ \quad \quad \quad \quad \quad \quad \quad \quad \;\; \times \exp \left( { - i\left( {k\frac{{r^{2} }}{{2R\left( {z,\omega } \right)}} + \ell \theta + kz - \psi \left( {z,\omega } \right)} \right)} \right) \hfill \\ \end{gathered} $$

where ${L}_{p}^{\left| \ell\right|}$ is the generalized Laguerre polynomial, ${C}_{ \ell,p}^{LG}$ is the required normalization constant, $w\left(z\right)$ is the beam size at a distance of *z*, $R\left(z,\omega \right)=z(1+{({z}_{R}(\omega )/z)}^{2})$ is the radius of curvature of the beam's wavefronts at *z*, where ${z}_{R}(\omega )$ is the Rayleigh range, $k$ is the wave number, $\omega$ is the angular frequency, $\left(r,\theta ,z\right)$ represents the cylindrical coordinate, and $\psi \left(z\right)$ is the Gouy phase and equals $\left(\left| \ell\right|+2p+1\right)\mathrm{arctan}\left(z/{z}_{R}(\omega )\right)$;

The electrical fields of the limited-size aperture- and multipath-affected OAM beams are generated by numerically truncating and translating the ideal OAM fields at a given propagation distance of *z* = *L*.

Subsequently, the direct field is truncated by the circular receiver aperture, and can be denoted as

2 $$ E_{direct field} = LG_{\ell ,0} \left( {x,y} \right) \cdot Aperture\left( {x,y} \right) $$

where ${LG}_{ \ell,0}$ is the OAM field emitted by the transmitter at the distance of *L*, $$Aperture\left( {x,y} \right) = \left\{ {\begin{array}{*{20}c} {0, \sqrt {x^{2} + y^{2} } < r_{receiver} } \\ {1, \sqrt {x^{2} + y^{2} } \ge r_{receiver} } \\ \end{array} } \right.$$ is the aperture function at the receiver, and ${r}_{receiver}$ is the radius of the receiver aperture.

The schematic of the reflected field can be referred to Figs. 1 and 5. The reflected field is given as an OAM $- \ell$ transmitted from an image transmitter with a lateral translation of 2 h from the original Tx. The field below the reflector is thus truncated. The mathematic expression of the reflected field is given as

3 $$ E_{reflected field} = LG_{ - \ell ,0} \left( {x,y + 2h} \right) \cdot Truncation\left( {x,y} \right) \cdot Aperture\left( {x,y} \right) $$

where ${LG}_{- \ell,0}(x,y+2h)$ is the OAM $- \ell$ field with a lateral translation of 2 h (the reflector is assumed to be placed along the *y*-axis), $$Truncation\left( {x,y} \right) = \left\{ {\begin{array}{*{20}c} {0, y < 0} \\ {1, y \ge 0} \\ \end{array} } \right.$$ is the truncation caused by the reflector. The electrical field below the reflector is truncated. In our simulation, we assume the reflector distance *h* > ${r}_{receiver}$.

For the multipath effect, the electrical field of the total path is the coherent sum of the direct and reflected fields

4 $$ E_{total field} = E_{direct field} + E_{reflected field} $$

The normalized 2-D LG spectrum is calculated to analyze the received beam. Each point of the spectrum is defined as the power weight coefficient of the LG mode with order *ℓ* and *p*, given as^3^

5 $$ |C_{\ell ,p} |^{2} = \left| {{\iint }E_{1} \left( {x,y} \right)E_{2}^{*} \left( {x,y} \right)dxdy } \right|^{2} $$

where ${E}_{1}\left(x,y\right)$ is the normalized electrical field of the received beam and ${E}_{2}\left(x,y\right)$ is the normalized electrical field of a pure ${\mathrm{LG}}_{ \ell,p}$ beam with the same beam waist ${w}_{0}$ as ${E}_{1}.$ The propagation distance for both ${E}_{1}$ and ${E}_{2}$ is $L$.

To calculate the 2-D LG spectrum, we vary the modal indices of $\ell$ from − 10 to 10 and *p* from 0 to 20. For each LG mode, we calculate the normalized modal coefficient ${{|C}_{ \ell,p}|}^{2}$ as the modal component on each mode.

The received power of certain ${LG}_{ \ell,p}$ mode is given as

6 $$ Power = |C_{\ell ,p} |^{2} \left| {{\iint }E_{total field} \cdot E_{total field}^{*} } \right|^{2} $$

where the integral $\left| {{\iint }E_{total field} \cdot E_{total field}^{*} } \right|^{2}$ represents the normalized total received power of the beam considering the truncation caused by the received aperture. Subsequently, the multiplication of the modal coefficient and total received power gives the normalized received power on each mode.

### Assumptions

For the convenience of analysis, the following assumptions are made:

(i) Atmospheric absorption loss is not considered for simplicity, such that only the degradation due to modal power coupling is considered in our simulation. In general, the atmospheric loss also degrades the performance of a real-life THz link^53^;
(ii) The diameter of the input OAM beam with OAM order *ℓ* is calculated by $D=2{w}_{0}\sqrt{\left| \ell\right|+1}$ where ${w}_{0}$ is the waist at a distance of 0^45^. In this paper, the aperture radius *r* is assumed to be the largest radius of the transmitted OAM beams (e.g., $r=2{w}_{0}$ for OAM ± 3, $r=\sqrt{6}{w}_{0}$ for OAM ± 5);
(iii) The transmitter and receiver aperture radii *r* are chosen to always be smaller than the reflector distance *h*.

### Details of parameter definitions

The parameters used in the model are listed in Table 1.

**Table 1:** Parameters.

*f*	Carrier frequency of the transmitted beam
*w*_0_	Beam waist of the transmitted beam
*r*	The radius of the transmitter and receiver aperture
*ℓ*	Azimuthal LG modal indices
*p*	Radial LG modal indices
*h*	Reflector distance
*L*	Propagation distance

## Data Availability

All data, theory details, simulation details that support the findings of this study are available from the corresponding authors on reasonable request.
